# New Species of *Boletellus* Section *Boletellus* (Boletaceae, Boletales) from Japan, *B*. *aurocontextus* sp. nov. and *B*. *areolatus* sp. nov.

**DOI:** 10.1371/journal.pone.0128184

**Published:** 2015-06-17

**Authors:** Hirotoshi Sato, Tsutomu Hattori

**Affiliations:** 1 Center for Ecological Research, Kyoto University, 509–3, 2-chome, Hirano, Otsu, Shiga, 520–2113, Japan; 2 Forestry and Forest Products Research Institute, Matsunosato 1, Tsukuba, Ibaraki, 305–8687, Japan; University of Florida, UNITED STATES

## Abstract

We describe and illustrate two new species of *Boletellus* section *Boletellus*, *B*. *aurocontextus* sp. nov. and *B*. *areolatus* sp. nov., which are generally assumed to be *B*. *emodensis*. In this study, we reconstructed separate molecular phylogenetic trees of section *Boletellus* using the nucleotide sequences of the internal transcribed spacer (ITS) region of nuclear ribosomal DNA, the largest subunit (RPB1) and the second-largest subunit (RPB2) of nuclear RNA polymerase II gene and mitochondrial cytochrome oxidase subunit 3 (*cox3*) gene. We also examined the morphologies of *B*. *emodensis* sensu lato (s.l.) and other related species for comparison. The molecular phylogenetic tree inferred from the sequences of nuclear DNA (ITS, and combined dataset of RPB1 and RPB2) indicated that three genetically and phylogenetically well-separated lineages were present within *B*. *emodensis* s.l. These three lineages were also distinguished on the basis of the molecular phylogenetic tree constructed using the sequences of mitochondrial DNA (*cox3*), suggesting distinct cytonuclear disequilibria (i.e., evidence of reproductive isolation) among these lineages. Therefore, these three lineages can be treated as independent species: *B*. *aurocontextus*, *B*. *areolatus*, and *B*. *emodensis*. *Boletellus aurocontextus* and *B*. *areolatus* are also distinct from *B*. *emodensis* by the macro- and microscopic morphologies. *Boletellus aurocontextus* is characterized by a pileus with bright yellow to lemon yellow context, which can be observed through a gap in the scales, and basidiospores with relatively large length (mean spore length, 21.4 μm; quotient of spore length and width, 2.51). In contrast, *B*. *areolatus* is characterized by a pileus with floccose to appressed thin scaly patches, a stipe with pallid or pale cream color at the upper half, and basidiospores with relatively small length (mean spore length, 16.5 μm; quotient of spore length and width, 1.80).

## Introduction

The genus *Boletellus* was originally described by Murrill in the family Boletaceae [1], and ca. 50 species of this genus have since been described worldwide [2]. *Boletellus* is an ectomycorrhizal fungus that forms a mutualistic relationship with host trees [3], although some species of this genus are often habitat on tree stumps or rotten wood. It is usually characterized by a yellow hymenophore and olive brown elongate to fusoid basidiospores with longitudinally winged basidiospores [4,5]. Seven sections were introduced in this genus by Singer [5], including those characterized by longitudinally winged spores (sections *Boletellus*, *Chrysenteroidei*, *Ixocephali*, and *Dictyopodes*), smooth spores (section *Mirabilis*), spores with imbedded short spines (section *Allospori*), and reticulate spores (section *Retispori*). Recent molecular phylogenetic studies indicate that *Boletellus* is polyphyletic [6–8] and thus the definition of this genus remains controversial.

*Boletellus* section *Boletellus*, in which the type species of the genus, i.e., *B*. *ananas* (M.A. Curtis) Murrill, is included, is one of the most well-defined sections among those defined by Singer [5]. This section unites taxa those have dry, reddish-pink to vinaceous-purple pileus finely covered with floccose or squamose scales and elongate to fusoid basidiospores with longitudinally winged ridges. Several species in this section have been reported from tropical areas of Southeast Asia [9], Central America [10,11], the warm temperate and subtropical areas of East Asia [12–15], North America [10], as well as Australia [16]. However, only a single species *B*. *emodensis* (Berk.) Singer, in this section was reported from Japan [12,13]; this species was first described as *B*. *floriformis* Imazeki [17], but later synonymized with *B*. *emodensis* [9].

Notably, several distinct morphological variations of *B*. *emodensis* have been reported [12,14]. Among those identified as *B*. *emodensis* in Japan [12], some specimens were clearly characterized by a pileus covered with relatively small scales, showing yellowish context through a gap in the scales. Moreover, distinct variations in basidiospores length and width have been reported within this species [14]. Therefore, we hypothesized that the species currently named as *B*. *emodensis* actually represents a complex composed of several different species.

In the present study, we reconstructed molecular phylogenetic trees using nucleotide sequences of both nuclear and mitochondrial DNA from *B*. *emodensis* s. l. and the related species to detect genetically and phylogenetically separated lineages within *B*. *emodensis* s. l. We also compared the morphological features of *B*. *emodensis* s. l. and the holotypes of related species, including *B*. *ananas*, *B*. *dissiliens* (Corner) Pegler & Young, *B emodensis*, and *B*. *paradoxus* (Massee) E.-J. Gilbert.

## Materials and Methods

### Field survey

From July 9 to August 24, 2009, 132 specimens of *B*. *emodensis* s. l. were collected from mixed forests of *Castanopsis cuspidata* and evergreen *Quercus* spp. and mixed forests of *Pinus densiflora* and deciduous *Quercus* spp. in Honshu and Kyushu, Japan. In addition, one specimen of *B*. cf. *paradoxus* was collected from lowland, mixed-dipterocarp forest in the Lambir Hills National Park, Sarawak, Borneo, on January 19, 2011. These specimens are deposited in the National Museum of Nature and Science, Tokyo (TNS).

We state that no specific permits were required for the described field studies in Japan, and the location was not privately-owned or protected in any way. Moreover, field study in the Lambir Hills National Park was conducted in accordance with a Memorandum of Understanding signed between the Sarawak Forestry Corporation and the Japan Research Consortium for Tropical Forests in Sarawak in November 2005. These field studies did not target endangered or protected species.

### DNA extraction, PCR amplification, and sequencing

Total DNA was extracted from the tissue of voucher specimens listed in Table 1 using a cetyltrimethylammonium bromide (CTAB) method, as described previously [18]. The internal transcribed spacer (ITS) region of nuclear ribosomal DNA was amplified by PCR using the universal primers ITS1 and ITS4 [19]. For the amplifications of the largest subunit (RPB1) and the second-largest subunit (RPB2) of nuclear RNA polymerase II region, primer pairs RPB1-B-F/RPB1-B-R and RPB2-B-F1/RPB2-B-R [8] were used, respectively. Partial sequences of mitochondrial cytochrome oxidase subunit 3 (*cox3*) gene were also amplified by PCR using the Boletales-specific primers COX3st-F forward and COXst-R reverse [18]. The amplification of these regions was performed in a total 10-μL reaction mixture containing 1× Ampdirect buffer with dNTPs, 5 pmol of both forward and reverse primers, 0.5 U of BIOTAQ Hot Start DNA Polymerase (Shimazu, Kyoto), and 10–50 ng of total DNA. Cycling parameters for PCR: hot start at 95C for 10 min; followed by 40 cycles at 95C for 30 s, at 50–55C for 30 s, and at 72C for 60 s; and a final extension at 72C for 7 min. Before nucleotide sequencing, PCR products were purified using ExoSAP-IT (GE Healthcare) according to the manufacturer’s instructions. The purified PCR products were sequenced using the same primers that were used for amplification. For ITS region, the universal primers ITS2 and ITS3 [19] were also used as internal primers for sequencing. Nucleotide sequencing was performed using an ABI 3130 automated sequencer (Applied Biosystems, Foster City, CA) with BigDye Terminator v3.1 Cycle Sequencing Kit (Applied Biosystems), following the manufacturer’s instructions.

**Table 1:** Voucher sample information and GenBank accession number of sequence data used in this study.

Voucher Sample Information		GenBank Accession			
Taxa	Herbarium ID	ITS	RPB1	RPB2	*cox3*
*Boletellus* sec. *Boletellus*					
*B*. *emodensis*	TNS-F-61440	AB988989	AB999720	AB999753	AB989023
*B*. *emodensis*	TNS-F-61476	AB988999	AB999729	AB999762	AB989024
*B*. *emodensis*	TNS-F-61459	AB988992	AB999723	AB999756	AB989027
*B*. *emodensis*	TNS-F-61471	AB988995	AB999725	AB999758	AB989028
*B*. *emodensis*	TNS-F-61494	AB989001	AB999731	AB999764	AB989029
*B*. *emodensis*	TNS-F-61495	AB989002	AB999732	AB999765	AB989030
*B*. *emodensis*	TNS-F-61496	AB989003	AB999733	AB999766	AB989031
*B*. *emodensis*	TNS-F-61547	AB989012	AB999742	AB999775	AB989047
*B*. *emodensis*	TNS-F-61549	AB989013	AB999743	AB999776	AB989048
*B*. *emodensis*	TNS-F-61564	AB989019	AB999749	AB999782	AB989053
*B*. *emodensis*	TNS-F-61472	AB988996	AB999726	AB999759	AB989043
*B*. *aurocontextus*	TNS-F-61460	AB988993	AB999724	AB999757	AB989032
*B*. *aurocontextus*	TNS-F-61474	AB988997	AB999727	AB999760	AB989033
*B*. *aurocontextus*	TNS-F-61478	AB989000	AB999730	AB999763	AB989034
*B*. *aurocontextus*	TNS-F-61498	AB989004	AB999734	AB999767	AB989035
*B*. *aurocontextus*	TNS-F-61500	AB989006	AB999736	AB999769	AB989036
*B*. *aurocontextus*	TNS-F-61501	AB989007	AB999737	AB999770	AB989037
*B*. *aurocontextus*	TNS-F-61504	AB989010	AB999740	AB999773	AB989038
*B*. *aurocontextus*	TNS-F-61552	AB989014	AB999744	AB999777	AB989039
*B*. *aurocontextus*	TNS-F-61467	AB988994			AB989040
*B*. *aurocontextus*	TNS-F-61502	AB989008	AB999738	AB999771	AB989041
*B*. *aurocontextus*	TNS-F-61505	AB989011	AB999741	AB999774	AB989042
*B*. *aurocontextus*	TNS-F-61553	AB989015	AB999745	AB999778	AB989049
*B*. *aurocontextus*	TNS-F-61475	AB988998	AB999728	AB999761	AB989044
*B*. *aurocontextus*	TNS-F-61499	AB989005	AB999735	AB999768	AB989045
*B*. *aurocontextus*	TNS-F-61503	AB989009	AB999739	AB999772	AB989046
*B*. *aurocontextus*	TNS-F-61559	AB989016	AB999746	AB999779	AB989050
*B*. *aurocontextus*	TNS-F-61562	AB989018	AB999748	AB999781	AB989052
*B*. *aurocontextus*	TNS-F-61566	AB989020	AB999750	AB999783	AB989054
*B*. *areolatus*	TNS-F-61444	AB988990	AB999721	AB999754	AB989025
*B*. *areolatus*	TNS-F-61449	AB988991	AB999722	AB999755	AB989026
*B*. *areolatus*	TNS-F-61560	AB989017	AB999747	AB999780	AB989051
*B*. *areolatus*	TNS-F-61568	AB989021	AB999751	AB999784	AB989055
*B*. *cf*. *paradoxus*	TNS-F-61570	AB989022	AB999752	AB999785	
B. ananas*	TH8819		HQ161822		
B. ananas*	TH6264	JN168685			
B. aff. emodensis*	HKAS_52678		KF112621	KF112757	
B. sp.*	HKAS-58713		KF112623	KF112759	
B. sp.*	HKAS-59536		KF112622	KF112758	
Outgroups					
Aureoboletus sp.*	GDGM32601	KF265358			
A. thibetanus*	MAK-ar001				AB426530
Heimioporus japonicus*	MAK-h003				AB426531
H. japonicus*	HKAS_52237		KF112618	KF112806	
Xerocomus sp.*	HKAS_76853		KF112635	KF112783	
X. aff. subtomentosus*	TRTC156926	JN021112			

* Nucleotide sequences were obtained from Genbank.

### Molecular phylogenetic analyses

After the sequences from the GenBank database were further added (Table 1), nucleotide sequences of the ITS region, RPB1, RPB2 and *cox3* genes were aligned using the multiple sequence alignment program, Muscle [20]. The aligned sequences of the ITS region, RPB1 and RPB2 genes were cleaned using Gblocks v0.91b [21], allowing smaller final blocks and gap positions within the final blocks. The resulting alignments of ITS, RPB1 RPB2 and *cox3* were deposited in the TreeBASE (Study ID: 17303; http://www.treebase.org/) and were separately subjected to molecular phylogenetic inference. According the previous report [8], closely related taxa of the ingroup were selected as outgroups (Table 1).

The most appropriate evolutionary model was determined for each dataset by comparing different evolutionary models via the corrected Akaike information criterion (AICc) [22,23] using the Kakusan 4 [24]. Phylogenetic inference based on the maximum-likelihood (ML) method was performed using TREEFINDER 2011-March version [25] with shotgun searches. Shotgun searches were repeated until no improvement was observed in the likelihood value. The confidence of the internal branches from the resulting tree was tested by bootstrap (BS) analysis [26] with 1,000 replications. Bootstrapping was also performed on the basis of the maximum parsimony method (10,000 replications) using PAUP 4.0b10 (PAUP*) phylogenetic inference package [27]. Pairwise ML distances were calculated using the TREEFINDER based on the most appropriate evolutionary model selected by Kakusan 4.

### Observation of morphological characteristics

To compare morphological characteristics, specimens of *B*. *emodensis* s. l. that were collected from warm temperate forests in Japan were examined. The holotypes of *B*. *ananas*, *B*. *dissiliens*, *B*. *emodensis*, and *B*. *paradoxus* were also examined for a comparative study of morphological features. Macro- and micro-morphological characteristics of basidiomes were described from fresh and dried specimens. The colors on the chart were composed of various percentages of the component colors: cyan, magenta, yellow, and black (CMYK). Microscopic observations were performed under an Eclipse 80i optical microscope (NIKON, Tokyo) with material (sections or fragments of the basidiome tissues) mounted in 5% potassium hydroxide (KOH) solution. Basidiospores measurements were performed at 1000× magnification under an Eclipse 80i optical microscope. The lengths and widths of 10 basidiospores were measured for each collection (for species with few available collections, 20 spores were measured per specimen). Mean and standard deviation (SD) of spore lengths and widths, and mean of the quotient of spore length and width (Q_m_) were then calculated for each species. Between-group differences in spore lengths and quotients of spore length and width (Q) were analyzed based on the Steel–Dwass multiple comparison procedure using R ver. 3.0.1 statistical software. Basidiospores were also observed under a scanning electron microscope (SEM) (Philips XL-series; Eindhoven) at a magnification of 5000×.

### Nomenclature

The electronic version of this article in Portable Document Format (PDF) in a work with an ISSN or ISBN will represent a published work according to the International Code of Nomenclature for algae, fungi, and plants, and hence the new names contained in the electronic publication of a PLOS ONE article are effectively published under that Code from the electronic edition alone, so there is no longer any need to provide printed copies.

In addition, new names contained in this work have been submitted to MycoBank, from where they will be made available to the Global Names Index. The unique MycoBank number can be resolved and the associated information viewed through any standard web browser by appending the MycoBank number contained in this publication to the prefix www.mycobank.org/MB. The online version of this work is archived and available from the following digital repositories: PubMed Central and LOCKSS.

## Results

### Molecular phylogenetic inference

**Fig 1:**
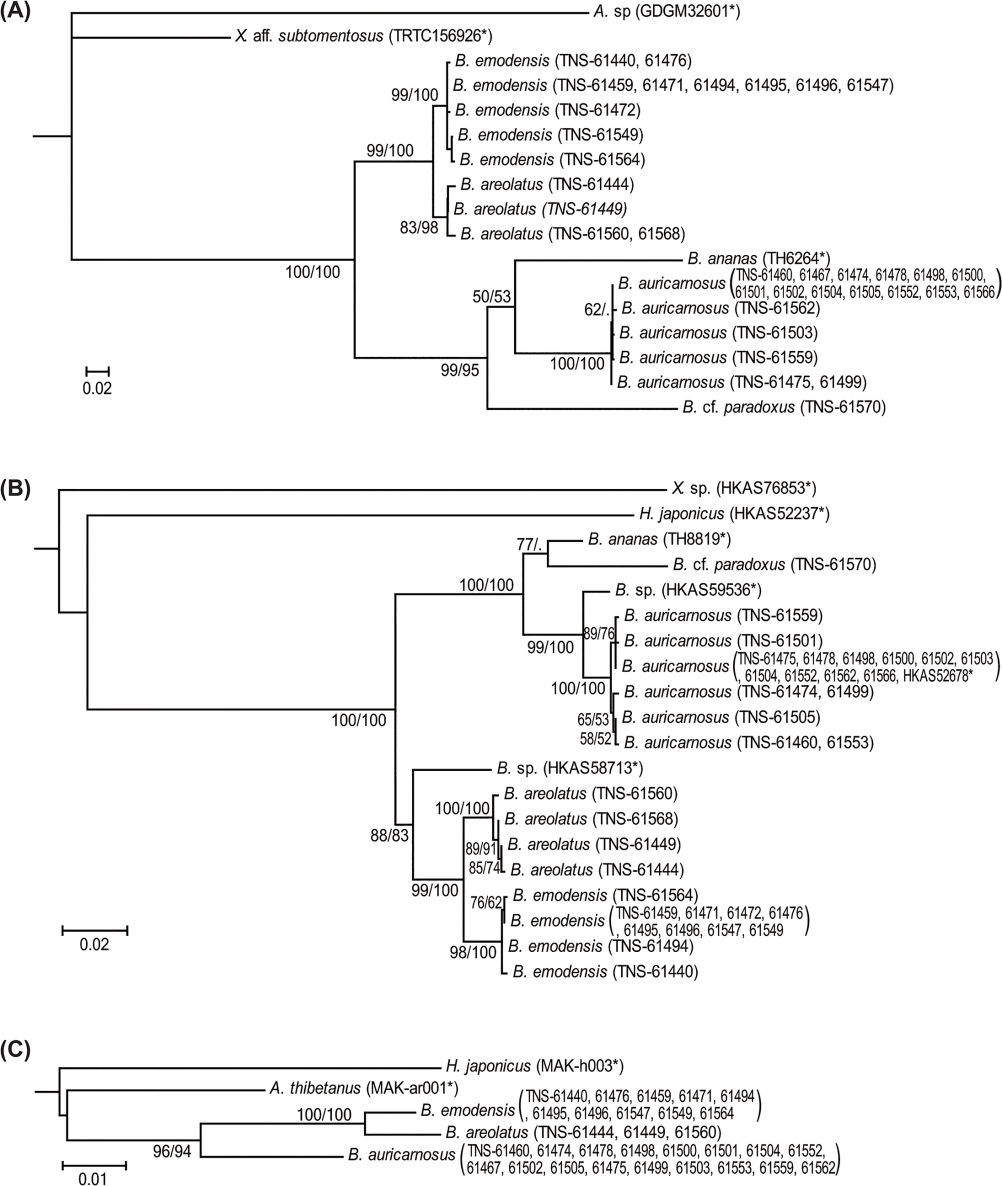
Maximum-likelihood tree of *Boletellus emodensis* s. l., as inferred from the nucleotide sequences of (A) the internal transcribed spacer (ITS) region, (B) combined dataset of the largest subunit (RPB1) and the second-largest subunit (RPB2) of RNA polymerase II gene and (C) cytochrome oxidase subunit 3 (*cox3*) gene. The numbers near the branches are bootstrap values determined by maximum-likelihood/parsimony analysis (>50%). An asterisk after voucher information indicates a sequence from GenBank.

The ITS dataset consisting 17 taxa of Boletaceae, included 1041 nucleotide sites for each taxon or sample, of which 252 were parsimony informative, after cleaning the aligned sequences using Gblocks. The most appropriate model for the ITS region determined using Kakusan 4 was the K80 + G model. The combined data of RPB1 and RPB2 (hereafter, RPB1-RPB2) comprised 20 taxa and 1338 total characteristics, of which 200 were parsimony informative, after cleaning the aligned sequences using Gblocks. For the RPB1-RPB2 data, K80, TN93, K80 and TIM+G were selected as the most appropriate models for the first, second, third codons and intron partition of RPB1 gene, whereas J1, F81 and K80+G were selected for the first, second and third codons of RPB2 gene (a codon proportional model was used). The *cox3* data set comprised five taxa and 571 total characteristics, of which 28 were parsimony informative. For the *cox3* gene, J2 + G, F81, and TVM models were selected as the most appropriate models for the first, second, and third codons (a codon proportional model was used). Topology of the ML trees based on ITS, RPB1-RPB2 and *cox3* sequences are shown in Fig 1A, 1B and 1C, respectively.

Based on the molecular phylogenetic trees inferred from the ITS sequences (Fig 1A), three genetically and phylogenetically separated lineages were confirmed within *B*. *emodensis* s. l. (hereafter, *B*. *emodensis*, *B*. *aurocontextus*, and *B*. *areolatus*). Small variations of the ITS sequences were observed within these three lineages: the mean ML distances within the *B*. *emodensis*, *B*. *aurocontextus* and *B*. *areolatus* were 0.0032, 0.0024, and 0.0075, respectively. Monophyly of each lineage was supported by high BS values (ML/MP BS: 99/100, 100/100, and 83/98, respectively; Fig 1A). The ML tree of the ITS region indicated that *B*. *emodensis* and *B*. *areolatus* formed a well-supported clade (ML/MP BS: 99/100), although these two lineages were genetically differentiated (mean ML distance: 0.0306). The phylogenetic tree also strongly supported the grouping of *B*. *ananas*, *B*. *aurocontextus*, and *B*. cf. *paradoxus* (ML/MP BS: 99/95), although these lineages were genetically separated.

The monophyletic clade of each lineage was also well supported in the ML tree inferred from RPB1-RPB2 sequences (ML/MP BS: 98/100, 100/100, and 100/100, respectively; Fig 1B). The mean ML distances of the RPB1-RPB2 sequences within the *B*. *emodensis*, *B*. *aurocontextus* and *B*. *areolatus* were 0.0015, 0.0025, and 0.0024, respectively. Moreover, a well-supported monophyletic clade was formed by *B*. *emodensis* and *B*. *areolatus* (ML/MP BS: 99/100), but these two lineages were genetically differentiated (mean ML distance: 0.0228). The monophyletic clade including *B*. *ananas*, *B*. *aurocontextus*, and *B*. cf. *paradoxus* was also found in the ML tree (ML/MP BS: 100/100).

Topology of the ML tree inferred from sequences of the *cox3* gene was almost concordant with those of the nuclear DNA (Fig 1C). No variations of the *cox3* sequences were observed within *B*. *emodensis*, *B*. *aurocontextus*, and *B*. *areolatus*. Notably, the three genetically separated lineages, i.e., *B*. *emodensis*, *B*. *aurocontextus*, and *B*. *areolatus*, were distinguished by *cox3* sequences, concordant with the results of the ITS and RPB1-RPB2 sequences (Fig 1A and 1B), although there were smaller genetic variations in the *cox3* sequences.

### Taxonomy

#### *Boletellus aurocontextus* Hirot. Sato, sp. nov. (Figs 2–4) [urn:lsid:mycobank.org:names: MB 810175]

Holotype: Japan. Kyoto Pref.: Kyoto, Higashiyama, Kiyomizu Hill, August 16, 2009 (TNS-F-61566).

Etymology: *aurocontextus*, Latin, golden context, referring to the yellowish context.

Diagnosis: *Boletellus aurocontextus* sp. nov. is characterized by the following unique characters: a pileus with bright yellow to lemon yellow context, which can be observed through a gap in the scales, and relatively large and elongated basidiospores, measuring 18.5–24.5 × 7.5–10 μm (Q_m_ = 2.51).

**Fig 2:**
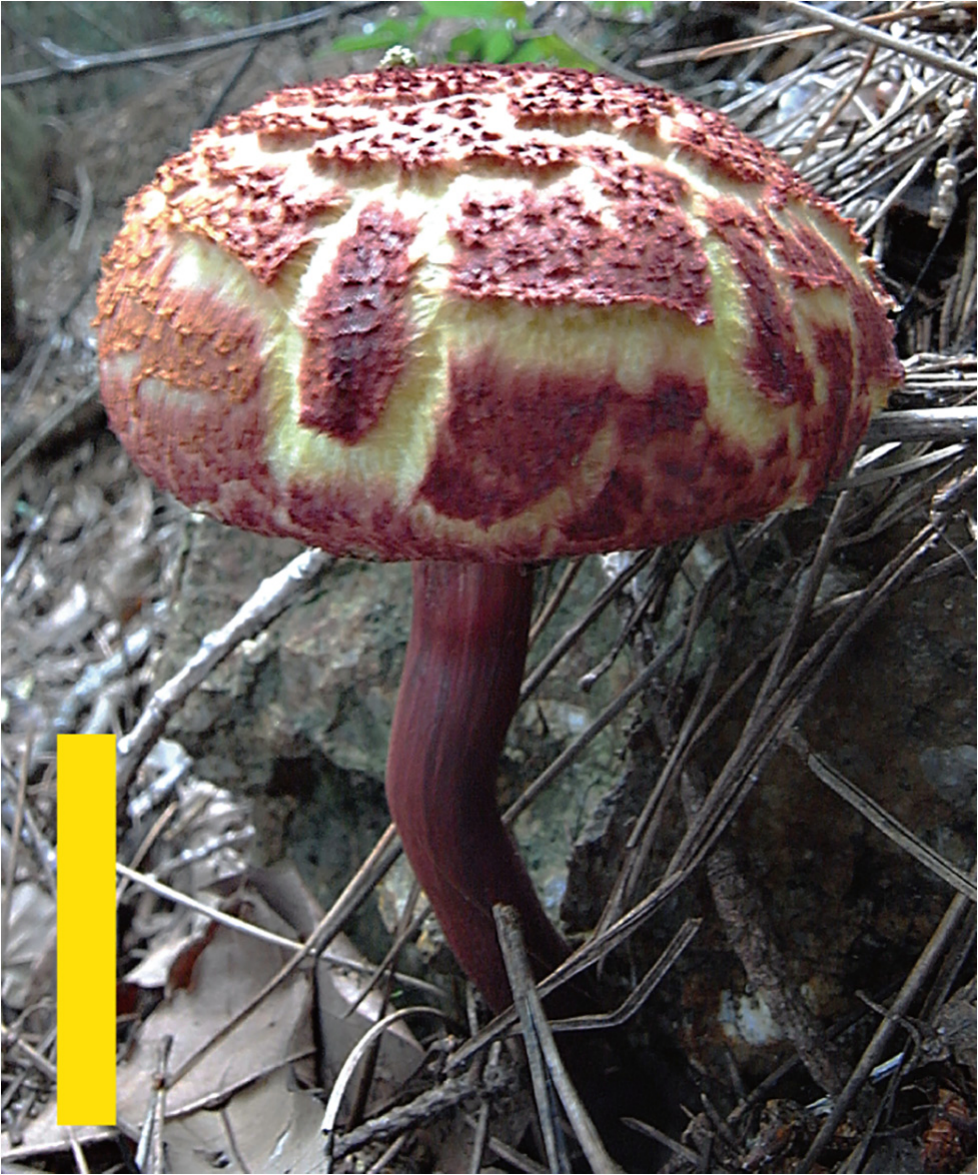
Basidiomes of *Boletellus aurocontextus* (TNS-F-61553). Bars: 5 cm.

**Fig 3:**
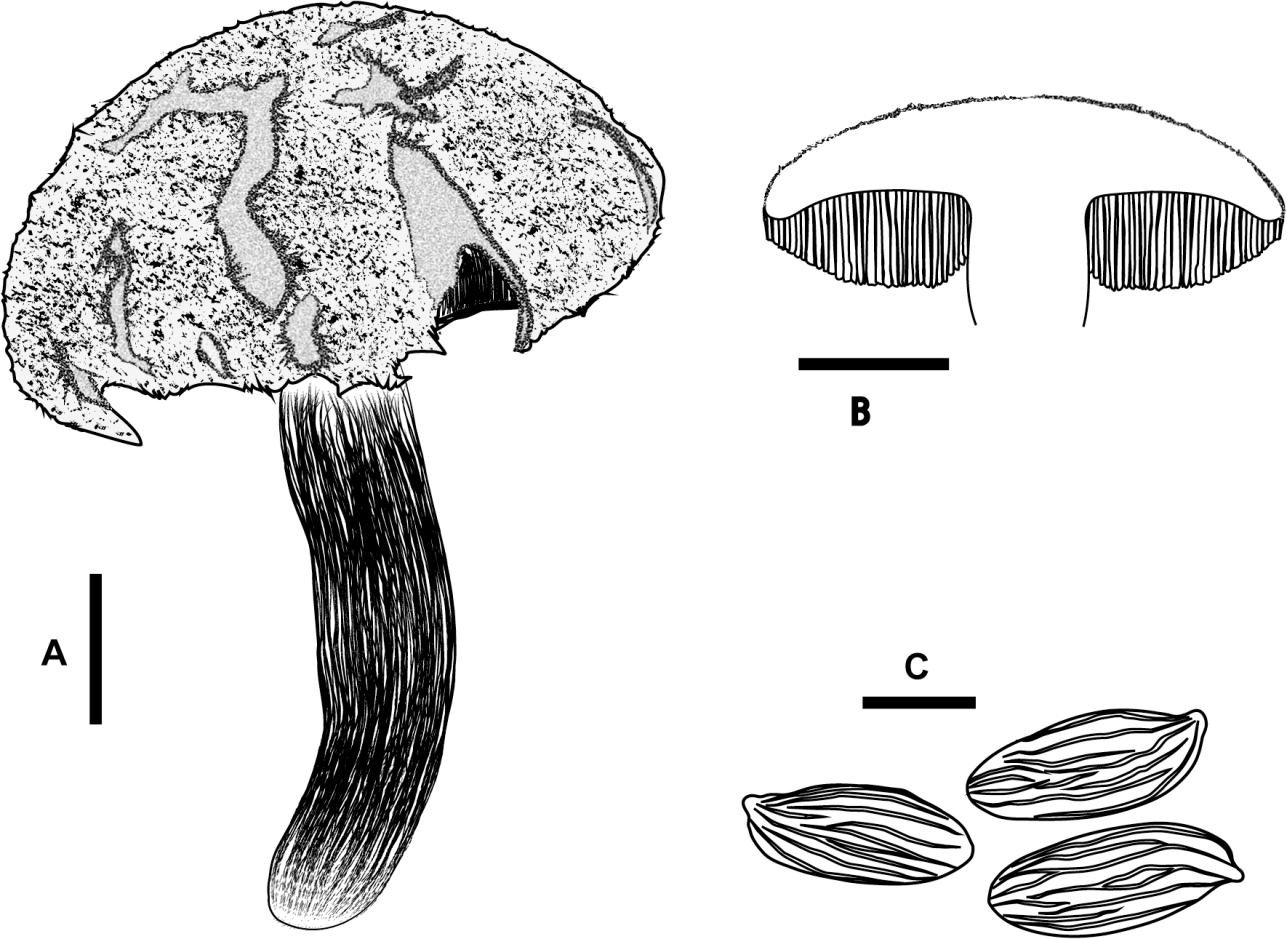
*Boletellus aurocontextus* (TNS-F-61566, holotype). (A) Basidiome. (B) Vertical section of basidiome. (C) Basidiospores. Bars A, B: 2 cm; C: 10 μm.

Description: Pileus 6–12 cm in diameter, at first convex then plano-convex, surface dry, finely covered with squamulose to verrucose scales, often rimulose to rimulose-areolate at maturity; scales up to 1.2 mm thick, rose-red (C: 35, M: 80, Y: 70, K: 0) to purplish red (C: 50, M: 90, Y: 50, K: 0), showing bright yellow (C: 10, M: 10, Y: 60, K: 0) to lemon yellow (C: 5, M: 0, Y: 45, K: 0) context through a gap of scales; margin widely appendiculate with a membranous veil concolorous with pileus surface (Figs 2 and 3A). Stipe 6–16 cm long, 8–16 mm thick, almost equal, straight or curved, longitudinally fibrillose, wine red (C: 40, M: 70 Y: 45, K: 0) to purple red (C: 60, M: 90, Y: 60, K: 25), often yellowish (C: 10, M: 5, Y: 40, K: 0) near the apex (Figs 2 and 3A). Tubes up to 15 mm long, sinuate, ventricose, yellow to mustard yellow (C: 20, M: 20, Y: 80, K: 0); pores up to 1 mm wide, concolorous with tubes (Fig 3B). Tubes and pores distinctly turning blue on bruising. Context of the pileus up to 15 mm thick in the center of the pileus, pale yellow (C: 10, M: 0, Y: 45, K: 0) to yellow (C: 10, M: 10, Y: 70, K: 0), turning blue (C: 90, M: 80, Y: 50, K: 0) when injured, but discoloration less distinct than in tubes. All parts of the basidiome more or less changing to blue (C: 90, M: 80, Y: 50, K: 0) then black (C: 90, M: 85, Y: 75, K: 65) on bruising. Spore-print fuscous-brown (C: 45, M: 65, Y: 90, K: 10).

**Fig 4:**
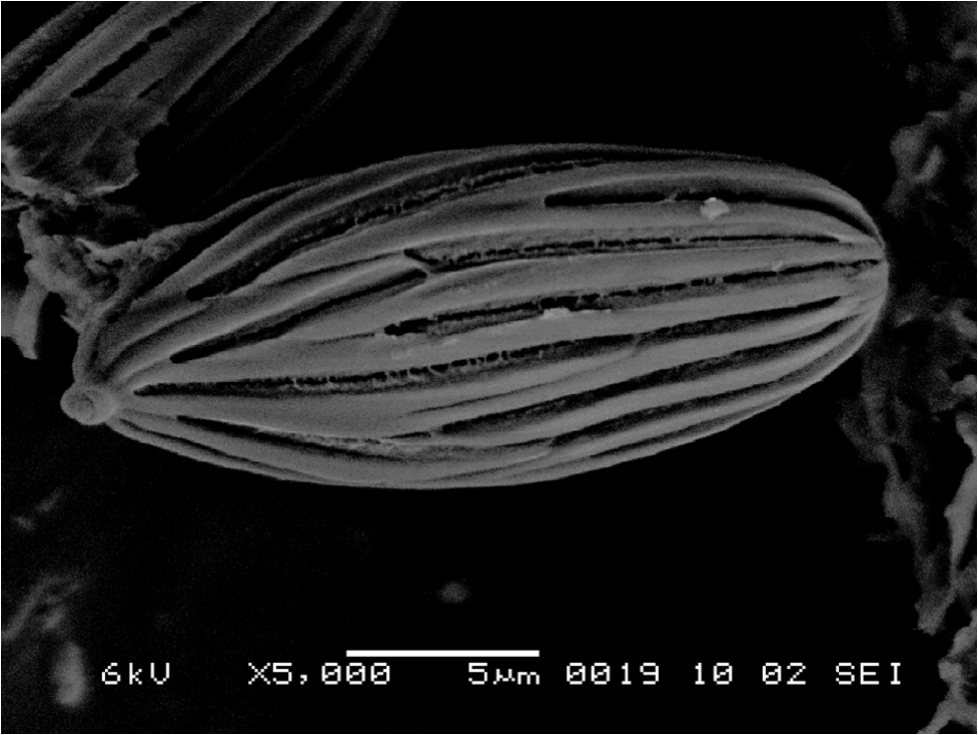
SEM of basidiospores of *Boletellus aurocontextus* (TNS-F-61566, holotype) at 5000×. Bars: 2 μm.

Basidiospores 18.5–24.5 × 7.5–10 μm (21.4 ± 1.3 × 8.5 ± 0.6; range, mean ± SD), Q_m_ = 2.51, including winged ornamentation, oblong ellipsoid to subcylindrical, dark brown to olivaceous brown in KOH solution, with the longitudinally winged ornamentations, 0.8–1.5 μm deep (Fig 3C and Fig 4). Basidia 26–41 × 8–14 μm, clavate, 4-spored; sterigmata 4–6 μm long. Cheilo- and pleurocystidia 8–18 × 28–52 μm, numerous, clavate, utriform to fusoid, hyaline or with brownishto fuscous contents, thin-walled. Scales of pileipellis composed of subradially arranged hyphae, 5–16 μm wide (terminal cells: 4–12 μm wide), cylindrical, reddish to fuscous brown. Pileus trama composed of interwoven hyphae, 7–12 μm wide. Hyphae of stipitipellis, cylindrical, 4–12 μm wide. Caulocystidia scattered, 30–90 × 8–20 μm, cylindrical, clavate, to fusiform. All hyphae without clamp connections.

Additional specimens examined: Japan. Kyoto Pref.: Kyoto, Sakyo, Yoshida-Hill, August 4, 2009 (TNS-F-61488, TNS-F-61493), August 11, 2009 (TNS-F-61511, 61512, 61515, 61518, 61519), August 23, 2009 (TNS-F-61567); Osaka Pref.: Mino, Mino park, July 22, 2009 (TNS-F-61461, 61462), July 31, 2009 (TNS-F-61469, 61473, 61474, 61475), August 5, 2009 (TNS-F-61498, 61499, 61500, 61501, 61502, 61503, 61504, 61505), August 12, 2009 (TNS-F-61552, 61553, 61554, 61555, 61556, 61557, 61558); Shiga Pref.: Otsu, Nagara Park, July 23, 2009 (TNS-F-61465); Shiga Pref.: Otsu, Ryukoku University August 1, 2009 (TNS-F-61468, 61478, 61479, 61480, 61481, 61482, 61483, 61484, 61485); Kanagawa Pref.: Kawasaki, Tama Ward, July 18, 2009 (TNS-F-61460); Chiba Pref.: Chosei, Chonan, Kasamori, August 16, 2009 (TNS-F-61559); Nara Pref.: Yamatokoriyama, Yatacho, August 15, 2009 (TNS-F-61562).

Habitat: Solitary or gregarious on the ground, tree stumps or rotten wood in mixed forests of *Pinus densiflora* and *Quercus serrata*. Putative ectomycorrhizal fungi.

Distribution: Honshu, Japan (presumably distributed in mixed conifer–broad-leaved forests in East Asia).

**Fig 5:**
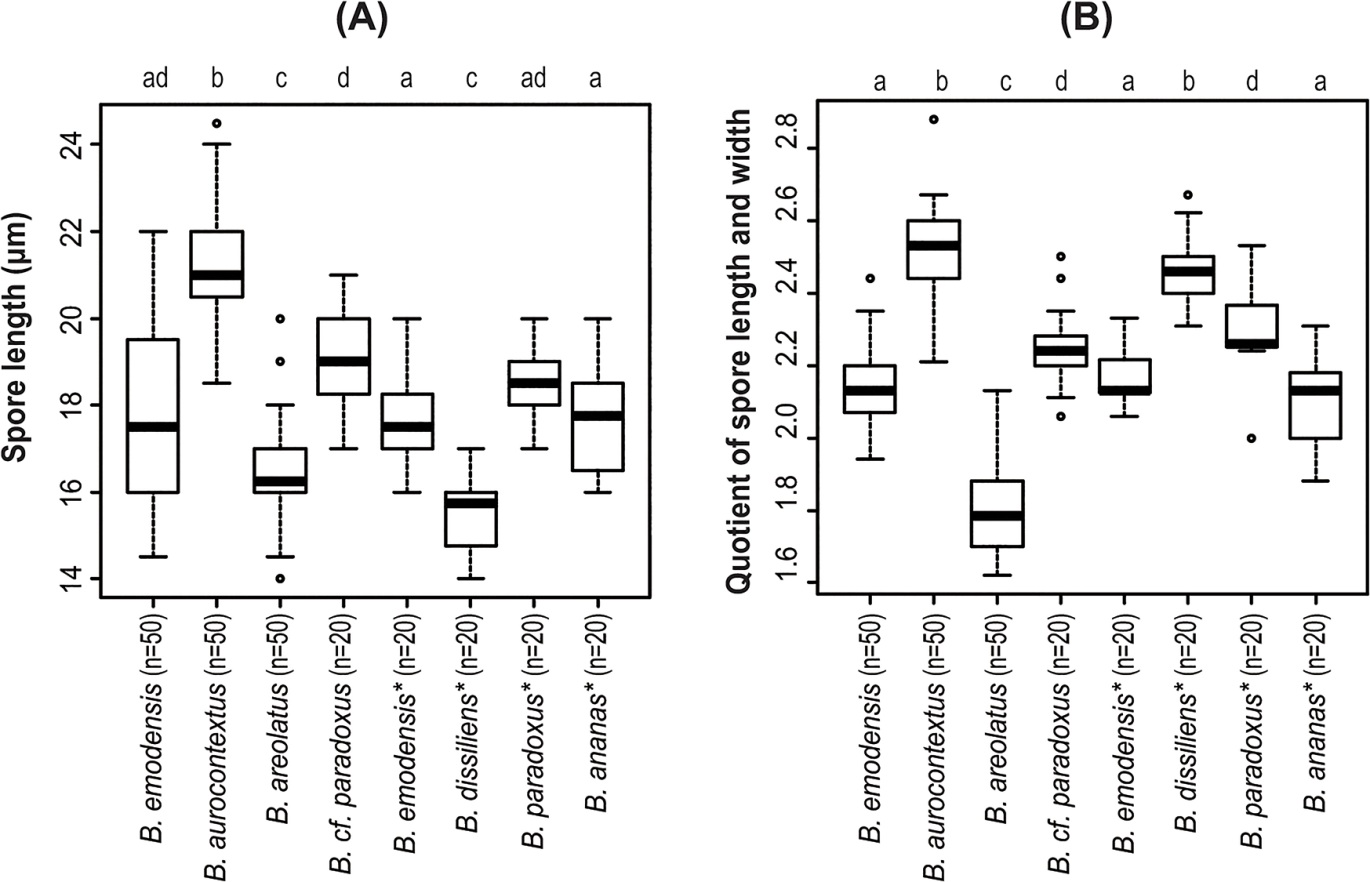
(A) Spore length and (B) quotient of spore length and width of *Boletellus emodensis* s. l. and the related species that were measured at 1000× magnification under an Eclipse 80i optical microscope. The box plot displays median (“bold line”), first and third quartile (“hinges”), and 95% confidence interval (“notches”). The number of spores examined is shown in parentheses. An asterisk after a species name indicates that spore measurement was performed only for the holotype. Different letters above the box plot (e.g., a, b, c, and d) indicate a statistically significant difference between groups.

Remarks: This species is distinguished from *B*. *emodensis* and other related species by the appearance of yellow or lemon yellow pileus context. This feature is particularly evident at the pileus surface on which the bright yellow to lemon yellow context can be observed through a gap in the scales. This species is also characterized by relatively large and elongated basidiospores measuring 18.5–24.5 × 7.5–10 μm (Fig 5A and 5B). Moreover, unlike *B*. *emodensis*, rose-red to purplish red coloration of the pileus scales likely persists throughout the stages of basidioma development. Large and elongated basidiospores of the present species are similar to those of *B*. *ananaeceps* (Berk.) Singer discovered in Australia, which has basidiospores measuring 16–26 × 6–11 μm [4]. However, the present species is distinguished from *B*. *ananaeceps* by the morphological feature of the pileus surface as described above. The lectotype of *B*. *annamiticus* (Pat.) E.-J. Gilbert (Basionym: *Strobilomyces annamiticus* Pat., 1909), which was discovered in Vietnam and is considered a synonym of *B*. *emodensis* [14,28], also has relatively large and elongated basidiospores measuring 21–24 × 7–8.5 μm, Q = 2.63–3.29 [14]. However, the lectotype is apparently too young to assess the spore size, and other important morphological characteristics of the present species have not been detected. Therefore, we conclude that the name “*B*. *annamiticus*” cannot be applied to the present species.

#### *Boletellus areolatus* Hirot. Sato, sp. nov. (Figs 6–8) [urn:lsid:mycobank.org:names: MB 810178]

Holotype: Japan. Miyazaki Pref.: Miyazaki, Hasugaike, August 24, 2009 (TNS-F-61568)

Etymology: *areolatus*, Latin, areolate, referring to the pileus surface with thin scaly patches.

Diagnosis: *Boletellus areolatus* sp. nov. is characterized by the following unique characters: a pileus with floccose to appressed thin scaly patches, a stipe with pallid or pale cream color at the upper half, and relatively small and broad basidiospores, measuring 14–20 × 7.5–11 μm (Q_m_ = 1.80).

**Fig 6:**
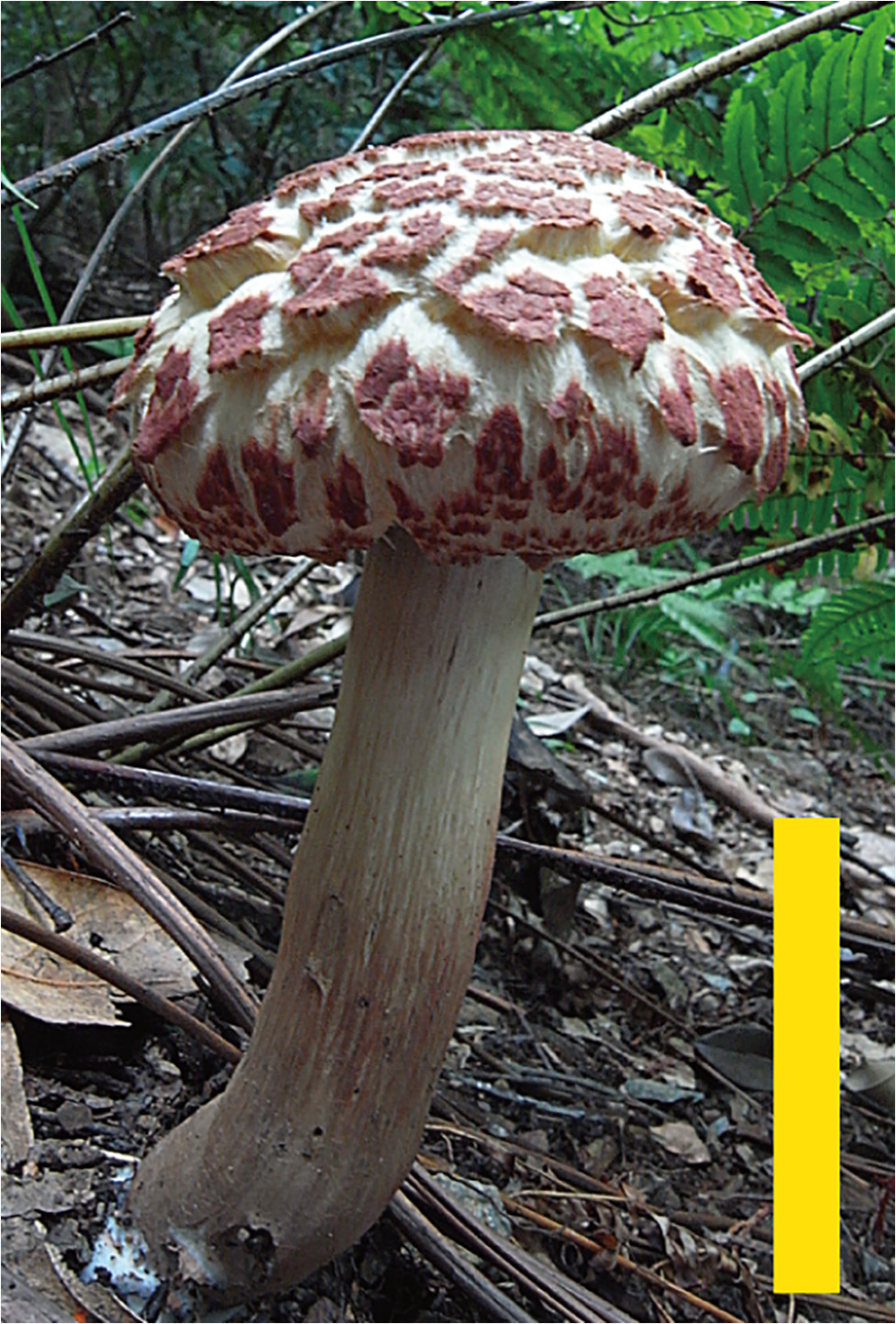
Basidiomes of *Boletellus areolatus* (TNS-F-61497). Bars: 5 cm.

**Fig 7:**
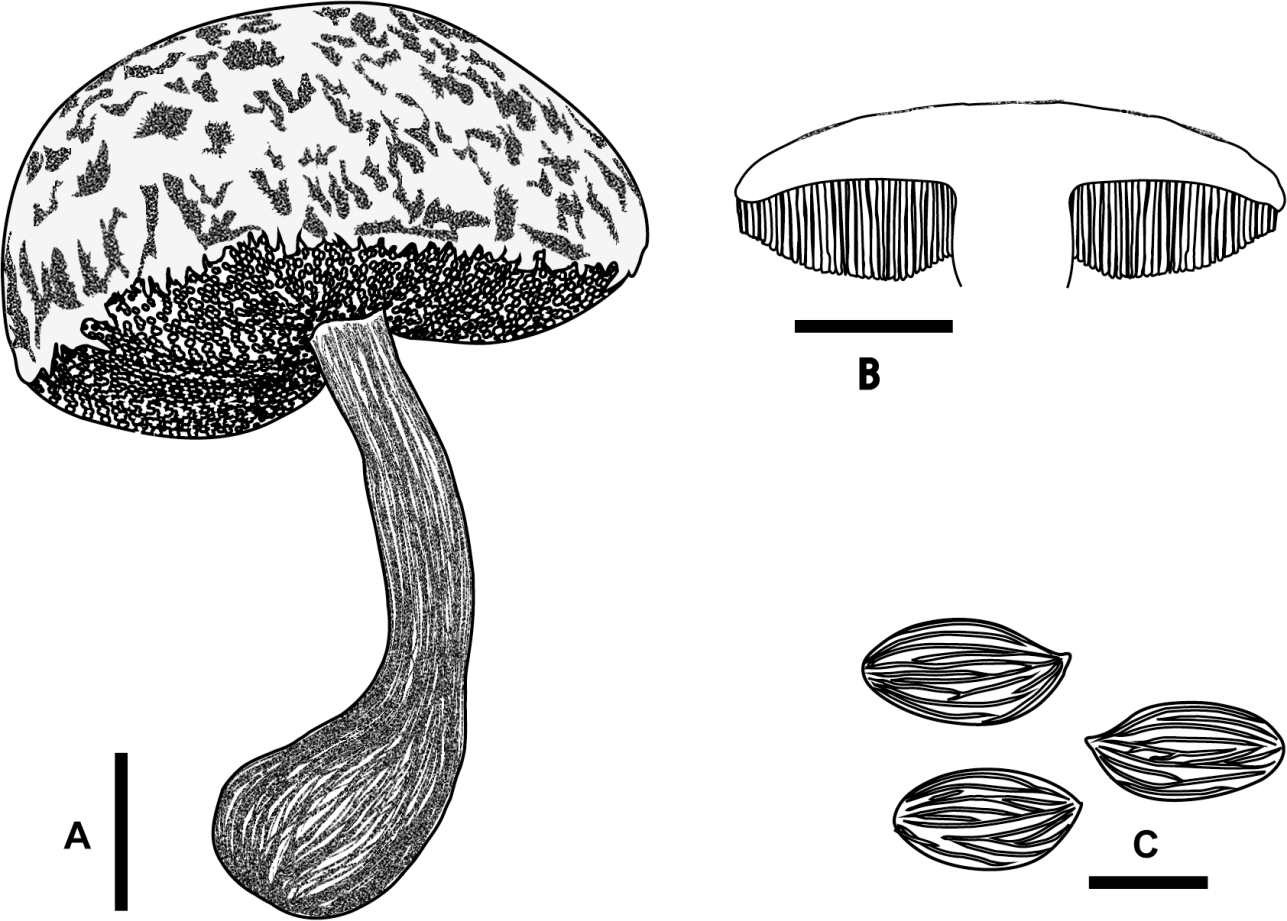
*Boletellus areolatus* (TNS-F-61568, holotype). (A) Basidiome. (B) Vertical section of basidiome. (C) Basidiospores. Bars: A, B: 2 cm; C: 10 μm.

**Fig 8:**
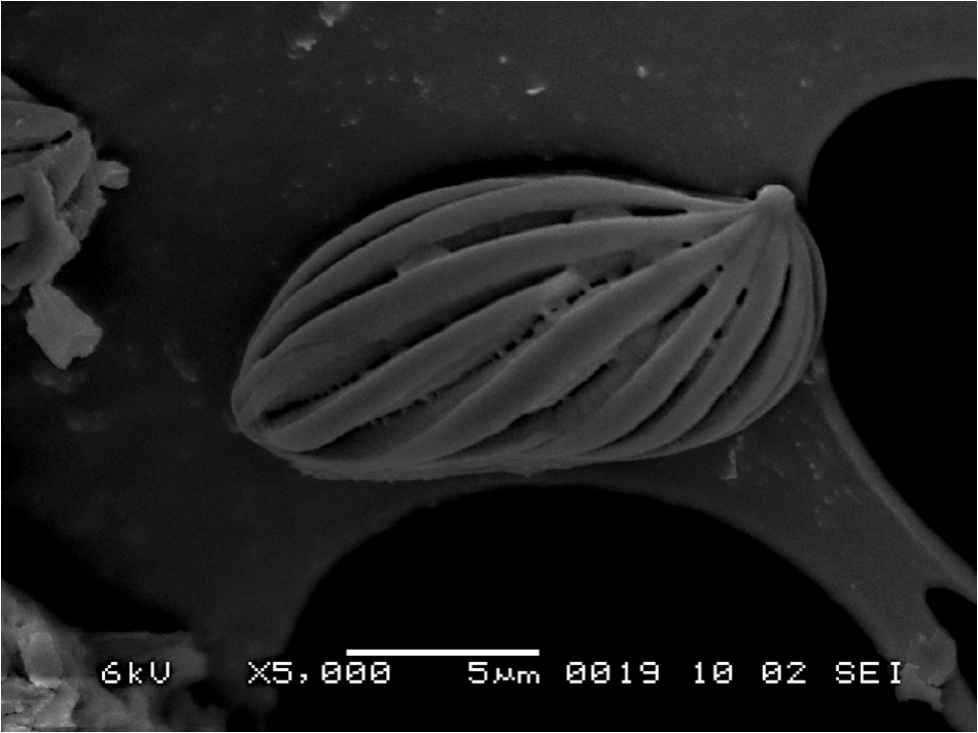
SEM of basidiospores of *Boletellus areolatus* (TNS-F-61568, holotype) at 5000×. Bars: 2 μm.

Description: Pileus 4–10 cm in diameter, at first convex then plano-convex, surface dry, at first tomentose to floccose with thin scaly patches, then coarsely cracking into large and small areas with tomentose, floccose to appressed thin scaly patches; scales up to 1 mm thick, at first pinkish red (C: 25, M: 40, Y: 35, K: 0) to brownish red (C: 45, M: 70, Y: 60, K: 0) then fading to yellowish brown (C: 25, M: 40, Y: 65, K: 0) to fuscous tan (C: 25, M: 25, Y: 50, K: 0) at maturity, showing pallid (C: 5, M: 0, Y: 2, K: 0) to pale cream (C: 10, M: 0, Y: 15, K: 0) context through a gap in the scales; margin widely appendiculate with a membranous veil concolorous with pileus surface (Figs 6 and 7A). Stipe 6–14 cm long, 8–18 mm thick, equal or subbulbous, straight or curved, longitudinally fibrillose, upper half pallid or pale cream (C: 10, M: 10, Y: 25, K: 0), lower half wine-red (C: 50, M: 75, Y: 50, K: 0) to dull rose-red (C: 40, M: 65, Y: 40, K: 0) (Fig 6). Tubes up to 15 mm long, sinuate, ventricose, mustard yellow (C: 20, M: 20, Y: 80, K: 0) to olive yellow (C: 40, M: 30, Y: 80, K: 0); pores up to 1 mm wide, concolorous with tubes (Fig 8B). Tubes and pores distinctly turning blue (C: 90, M: 80, Y: 50, K: 0) on bruising. Context of the pileus up to 12 mm thick in the center of the pileus, white (C: 2, M: 0, Y: 5, K: 0), turning blue (C: 90, M: 80, Y: 50, K: 0) when injured, but less distinct than in tubes. All parts of the basidiome more or less changing to blue (C: 90, M: 80, Y: 50, K: 0) then black (C: 90, M: 85, Y: 75, K: 65) on bruising. Spore-print fuscous-brown (C: 45, M: 65, Y: 90, K: 10).

Basidiospores 14–20 × 7.5–11 μm (16.5 ± 1.2 × 9.2 ± 0.8; range, mean ± SD), Q_m_ = 1.80, including winged ornamentation, ellipsoid, dark brown to olivaceous brown in KOH solution, with the longitudinally winged ornamentations, 0.8–1.4 μm deep (Fig 8C and Fig 8). Basidia 24–45 × 9–15 μm, clavate, 4-spored; sterigmata 4–6 μm long. Cheilo- and pleurocystidia 9–20 × 30–80 μm, numerous, clavate, utriform to fusoid, hyaline or with brownish to fuscous contents, thin-walled. Scales of pileipellis composed of subradially arranged hyphae, 5–15 μm wide (terminal cells: 5–14μm wide), cylindrical, reddish to fuscous brown. Pileus trama composed of interwoven hyphae, 5–20 μm wide. Hyphae of stipitipellis, cylindrical, 4–12 μm wide. Caulocystidia scattered, 32–63 × 8–15 μm, cylindrical, clavate, to fusiform. All hyphae without clamp connections.

Additional specimens examined: Japan. Miyazaki Pref.: Miyazaki, Hasugaike, August 24, 2009 (TNS-F-61569); Chiba Pref.: Chosei, Chonan, Kasamori August 16, 2009 (TNS-F-61560); Osaka Pref.: Mino, Mino-Park, July 15, 2009 (TNS-F-61444, 61449), August 5, 2009 (TNS-F-61497), August 12, 2009 (TNS-F-61550).

Habitat: Solitary or gregarious on the ground in mixed forests of *Castanopsis* spp. and evergreen *Quercus* spp.). Putative ectomycorrhizal fungi.

Distribution: Kyusyu and Honshu in Japan (presumably distributed in evergreen oak forests in East Asia).

Remarks: This species is morphologically similar to *B*. *emodensis* and *B*. *dissiliens*, but it shows a pileus with floccose to appressed thin scaly patches and a stipe with pallid or pale cream color at the upper half. This species is also characterized within the *Boletellus* section by smaller and broader basidiospores measuring 14–20 × 7.5–11 μm (Fig 5A and 5B).

#### *Related species*: *Boletellus emodensis* (Berk.) Singer, Annls Mycol. 40: 19 (1942) (Figs 9 and 10) = *Boletellus floriformis* Imazeki, Nagaoa 2: 42 (1952) [urn:lsid:mycobank.org:names: MB 472279]

**Fig 9:**
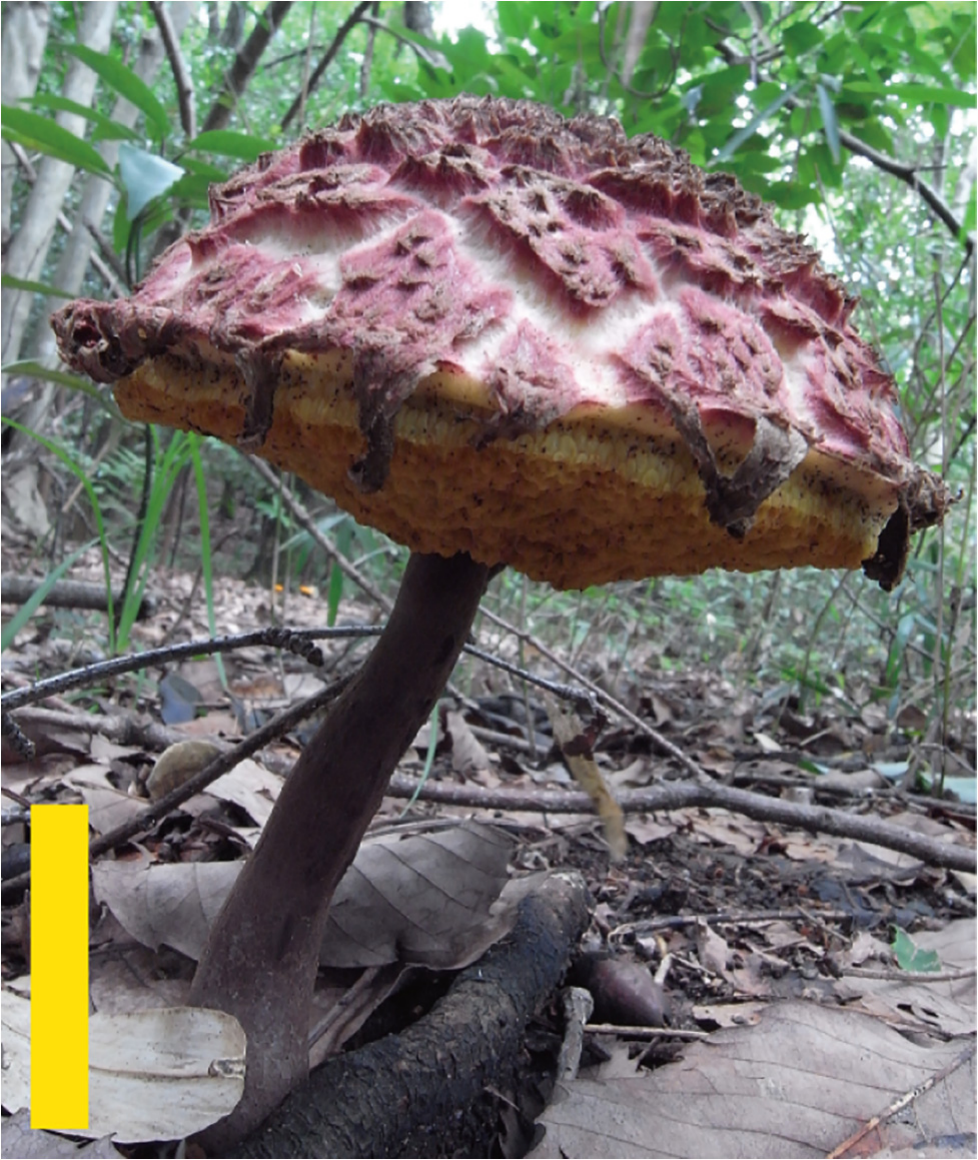
Basidiomes of *Boletellus emodensis* (TNS-F-61513). Bars: 5 cm.

Description: Pileus 6–14 cm in diameter, at first convex then plano-convex, surface dry, at first densely covered with thick floccose to squamulose scales then coarsely cracking into large and small areas with thick appressed to squamulose scales; scales up to 3.0 mm thick, sometimes recurved, at first bright purplish red (C: 55, M: 90, Y: 55, K: 0) to dull purplish red (C: 15, M: 50, Y: 10, K: 5) then fading to fuscous tan (C: 30, M: 40, Y: 50, K: 0) at maturity, showing pallid (C: 5, M: 5, Y: 5, K: 0) to pale yellow (C: 5, M: 5 Y: 10, K: 0) context through a gap in the scales; margin widely appendiculate with a membranous veil concolorous with pileus surface (Fig 9). Stipe 6–18 cm long, 10–20 mm thick, almost equal, straight or curved, longitudinally fibrillose, rose-red to fuscous red, sometimes yellowish near the apex (Fig 9). Tubes up to 18 mm long, sinuate, ventricose, mustard yellow (C: 20, M: 20, Y: 80, K: 0) to olive yellow (C: 40, M: 30, Y: 80, K: 0); pores up to 1 mm wide, concolorous with tubes. Tubes and pores distinctly turning blue (C: 90, M: 80, Y: 50, K: 0) on bruising. Context of the pileus up to 15 mm thick in the center of the pileus, white (C: 2, M: 0, Y: 5, K: 0), turning blue (C: 90, M: 80, Y: 50, K: 0) when injured, but less distinct than in tubes. All parts of the basidiome more or less changing to blue (C: 90, M: 80, Y: 50, K: 0) then black (C: 90, M: 85, Y: 75, K: 65) on bruising. Spore-print fuscous-brown.

**Fig 10:**
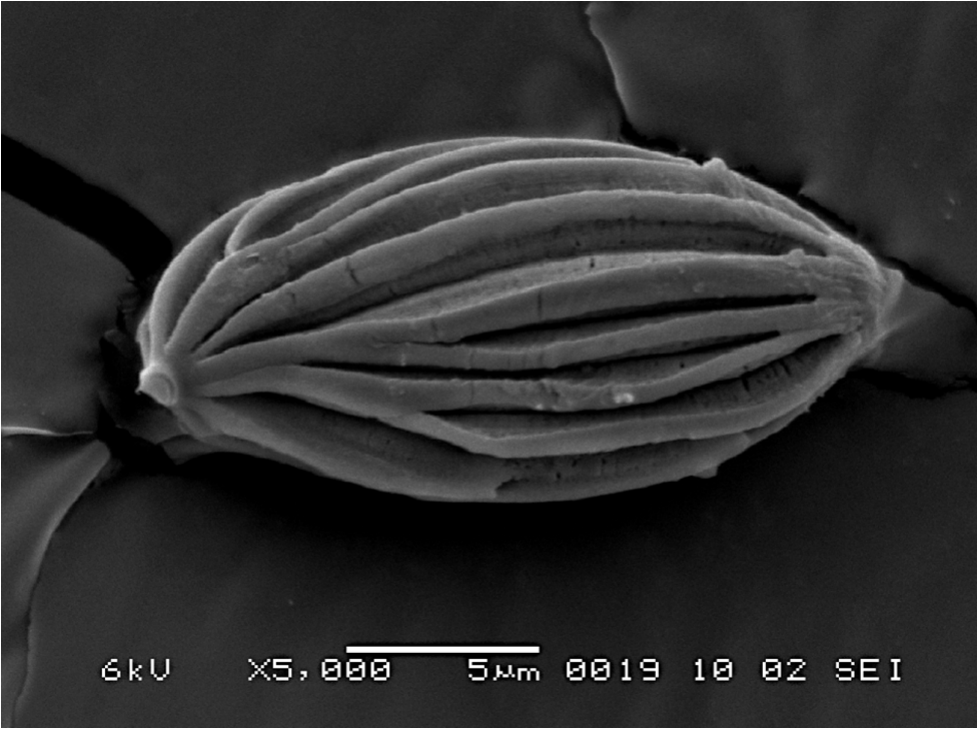
SEM of basidiospores of *Boletellus emodensis* (TNS-F-61459) at 5000×. Bars: 2 μm.

Basidiospores 14.5–22 × 6.5–10.5 μm (17.8±1.9 × 8.3 ± 0.8; range, mean ± SD), Q_m_ = 2.15, including ornamentation, oblong ellipsoid, dark brown to olivaceous brown in KOH solution, with the longitudinally winged ornamentations, 0.8–1.3 μm deep (Fig 10). Basidia 24–45 × 9–15 μm, clavate, 4-spored; sterigmata 4–6 μm long. Cheilo- and pleurocystidia 9–20 × 30–80 μm, numerous, clavate, utriform to fusoid, hyaline or with brownish to fuscous contents, thin-walled. Scales of pileipellis composed of subradially arranged hyphae, 5–15 μm wide (terminal cells: 6–20 μm), cylindrical, reddish to fuscous brown. Pileus trama composed of interwoven hyphae, 7–20 μm. Hyphae of stipitipellis, cylindrical, 4–12 μm wide. Caulocystidia scattered, 33–80 × 8–15 μm, cylindrical, clavate, to fusiform. All hyphae without clamp connections.

Species examined: India. Darjeeling (K(M): 178451, HOLOTYPE); Japan. Chiba Pref.: Chosei District, Chonan, Kasamori, August 16, 2009 (TNS-F-61561); Chiba Pref.: Kashiwa, Masuojoshi Park, July 9, 2009 (TNS-F-61439, TNS-F-61440); Hyogo Pref.: Akashi, Akashi Park, August 15, 2009 (TNS-F-61564); Kumamoto Pref.: Kumamoto, Mt. Tatsuta, July 14, 2009 (TNS-F-61441, 61442, 61443), July 15, 2009 (TNS-F-61455, 61456), August 8, 2009 (TNS-F-61506, 61507, 61508); Kyoto Pref.: Kyoto, Higashiyama, Kiyomizu Hill, July 26, 2009 (TNS-F-61466), August 16, 2009 (TNS-F-61565); Kyoto Pref.: Kyoto, Sakyo, Yoshida hill, August 4, 2009 (TNS-F-61489, 61490, 61491, 61492), August 11, 2009 (TNS-F-61509, 61510, 61513, 61514, 61516, 61517, 61520, 61521, 61522, 61523, 61524, 61525, 61526, 61527, 61528, 61529, 61530, 61531, 61532, 61533, 61534, 61535, 61536, 61537, 61538, 61539, 61540, 61541, 61542, 61543, 61544, 61545); Nara Pref.: Yamatokoriyama, Yatacho, August 15, 2009 (TNS-F-61563); Niigata Pref.: Myoko, July 29, 2009 (TNS-F-61486, 61487); Oita Pref.: Usa, July 19, 2009 (TNS-F-61457, 61458, 61459); Osaka Pref.: Mino, Mino Park, July 14, 2008 (TNS-F-61451, 61452, 61453), July 15, 2009 (TNS-F-61445, 61446, 61447, 61448), July 16, 2009 (TNS-F-61450, 61454), July 22, 2009 (TNS-F-61463, 61464), July 31, 2009 (TNS-F-61470, 61471, 61472, 61476, 61477), August 5, 2009 (TNS-F-61494, 61495, 61496), August 12, 2009 (TNS-F-61546, 61547, 61548, 61549, 61551); Shiga Pref.: Otsu, Nagara Park, July 13, 2009 (TNS-F-61438).

Habitat: Solitary or gregarious on the ground, tree stumps or rotten wood in mixed forests of *Castanopsis* spp. and evergreen *Quercus* spp. forests. Putative ectomycorrhizal fungi.

Distribution: Honshu and Kyushu in Japan, Darjeeling in India (presumably broadly distributed in evergreen oak forests in East Asia, Southeast Asia and South Asia).

Remarks: Transverse striae on the ribs of basidiospores, which had been considered as a key feature of this species [4,11], were not confirmed under the SEM in the present study. This characteristic was not detected in other related species examined in the present study, and we suggest that this is a dubious characteristic for *B*. *emodensis* and its related species within the genus *Boletellus*.

Although the holotype of *B*. *floriformis* was not traced in TFM, we concluded it as a synonym of *B*. *emodensis* because of the presence of the pallid to pale yellow context, stipe with rose-purple color at the upper half, and oblong ellipsoid basidiospores [17].

## Discussion

Based on the molecular phylogenetic tree inferred from nuclear ITS and RPB1-RPB2 sequences, we confirmed that *B*. *emodensis* s. l. is polyphyletic and separated into three independent clades that were genetically differentiated (Fig 1A and 1B). Although suggestive of only a small degree of genetic difference, the molecular phylogenetic tree inferred from the mitochondrial *cox3* sequences corresponded well to the results of the ITS and RPB1-RPB2 sequences (Fig 1A–1C). The distinct cytonuclear disequilibria observed among these three groups suggested that a reproductive barrier is present among these groups [29–32]. These results strongly support that these three genetic and phylogenetic lineages of *B*. *emodensis* s. l. be treated as different species: *B*. *emodensis*, *B*. *aurocontextus*, and *B*. *areolatus*. Moreover, the molecular phylogenetic trees inferred from the ITS, RPB1-RPB2 and *cox3* sequences suggest that *B*. *areolatus* is a sister group of *B*. *emodensis*, whereas *B*. *aurocontextus* is distantly related to *B*. *emodensis*.

*Boletellus aurocontextus* and *B*. *areolatus* are distinguished from *B*. *emodensis* and other related species by several morphological characteristics. In particular, the characteristics of the pileus surface, stipe, and basidiospores, as described above, are most distinctive among these species. For example, *B*. *aurocontextus* can be distinguished from other species of this section, including *B*. *ananaeceps* and *B*. *emodensis*, because of a pileus exhibiting a bright yellow to lemon yellow context through gaps in the scales and relatively large and elongated basidiospores. *Boletellus areolatus* is morphologically similar to *B*. *emodensis* particularly in coloration of the pileus surface, but the former is characterized by a pileus with flocosse to appressed thin scaly patches, a stipe with pallid or pale cream color at the upper half, and less elongated basidiospores. These characteristics are also useful to distinguish this species from *B*. *dissiliens*, which has relatively small basidiospores, as in this species.

Nucleotide sequences from specimens of *B*. *emodensis* collected in the type locality (India, Darjeeling) were not available, and thus there remains a possibility that the Japanese specimens identified as “*B*. *emodensis*” might be distinct from *B*. *emodensis*. Nevertheless, there are no reasonable grounds to distinguish them in the present state, because no significant morphological differences were detected between the Japanese specimens and the holotypes of this species. Moreover, evergreen oaks (e.g., *Quercus* and *Castanopsis*) were predominant in both of tropical upper montane forests around the type locality [33] and evergreen temperate forests in Japan, suggesting that potential host plants were presumably similar between these regions.

The geographical distribution of *B*. *aurocontextus*, *B*. *areolatus*, and *B*. *emodensis* may be restricted to temperate and subtropical regions of East Asia, Southeast Asia, and South Asia, as in case of most ectomycorrhizal fungal species that are observed in Japan [34]. *Boletellus aurocontextus* can be characterized by the distribution pattern and habitat. Our field survey implies that this species likely inhabits mixed forests of *Pinus* and deciduous *Quercus* species in Japan, although most species of the same section have been reported from broad-leaved evergreen forests in warm temperate, subtropical, and tropical areas [8,9]. However, further field surveys are required for revealing the distribution pattern and habitat (or host–fungus association) of this species.

### Key to the species of *Boletellus* section *Boletellus*

1. Q_m_ of spores < 2.4 ∙∙∙∙∙∙∙∙∙∙∙∙∙∙∙∙∙∙∙∙∙∙∙∙∙∙∙∙∙∙∙∙∙∙∙∙∙∙∙∙∙∙∙∙∙∙∙∙∙∙∙ 2
  Q_m_ of spores > 2.4 ∙∙∙∙∙∙∙∙∙∙∙∙∙∙∙∙∙∙∙∙∙∙∙∙∙∙∙∙∙∙∙∙∙∙∙∙∙∙∙∙∙∙∙∙∙∙∙∙∙∙∙∙ 5
2. Pileus covered with floccose to appressed thin scaly patches, basidiospores 14–20 × 7.5–11 μm (Q_m_ = 1.8) ∙∙∙∙∙∙∙∙∙∙∙∙∙∙∙∙∙∙∙∙∙∙∙∙∙∙∙∙∙∙∙∙∙∙∙∙∙∙∙∙∙∙∙∙∙∙∙∙∙∙∙∙∙∙∙∙∙∙∙∙∙∙∙∙∙∙∙∙∙∙∙∙∙∙∙∙∙∙∙∙ *B*. *areolatus*
  Pileus covered with thick appressed to squamulose scales, Q_m_ > 2.0 ∙∙∙∙∙∙∙∙∙∙∙∙∙∙∙∙∙∙∙∙∙∙∙∙∙∙∙∙∙∙∙∙∙∙∙∙∙∙∙∙∙∙∙∙∙∙∙∙∙∙∙∙∙∙∙∙∙∙∙∙∙∙∙∙∙∙∙∙∙∙∙∙∙∙∙∙∙∙∙∙∙∙∙∙∙∙∙∙∙∙∙∙∙∙∙∙∙∙∙∙∙∙∙∙∙ 3
3. Stipe white to pallid ∙∙∙∙∙∙∙∙∙∙∙∙∙∙∙∙∙∙∙∙∙∙∙∙∙∙∙∙∙∙∙∙∙∙∙∙∙∙∙∙∙∙∙∙∙∙∙∙∙∙∙∙∙∙∙∙∙∙∙∙∙∙∙∙∙∙∙∙ *B*. *ananas*
  Stipe red, umber or purple red ∙∙∙∙∙∙∙∙∙∙∙∙∙∙∙∙∙∙∙∙∙∙∙∙∙∙∙∙∙∙∙∙∙∙∙∙∙∙∙∙∙∙∙∙∙∙∙∙∙∙∙ 4
4. Pileus surface at first bright purplish red to dull purplish red then fading to fuscous tan, basidiospores 14.5–22 × 6.5–10.5 μm (Q_m_ = 2.15) ∙∙∙∙∙∙∙∙∙∙∙∙∙∙∙∙∙∙∙∙∙∙∙∙∙∙∙∙∙∙∙∙∙∙∙ *B*. *emodensis*
  Pileus surface more or less umber, basidiospores more elongated (17–20 × 7.5–8.5, Q_m_ = 2.30) ∙∙∙∙∙∙∙∙∙∙∙∙∙∙∙∙∙∙∙∙∙∙∙∙∙∙∙∙∙∙∙∙∙∙∙∙∙∙∙∙∙∙∙∙∙∙∙∙∙∙∙∙∙∙∙∙∙∙∙∙∙∙∙∙∙∙∙∙∙∙∙∙∙∙∙∙∙∙∙∙∙∙∙∙∙∙∙∙∙∙∙∙∙∙∙∙∙∙∙∙∙∙∙ *B*. *paradoxus*
5. Context of pileus bright yellow to lemon yellow (showing yellow to lemon yellow context through a gap of the scales), stipe wine red to purple red ∙∙∙∙∙∙∙∙∙∙∙∙∙∙∙∙∙∙∙∙∙∙∙∙∙∙∙∙ *B*. *aurocontextus*
  Context of pileus pallid, stipe pallid, pinkish tan or buff white ∙∙∙∙∙∙∙∙∙∙∙∙∙∙∙∙∙∙∙∙∙∙∙∙∙∙∙∙∙∙∙∙∙∙∙∙∙∙∙∙∙∙∙∙∙∙∙∙∙∙∙∙∙∙∙∙∙∙∙∙∙ ∙∙∙∙∙∙∙∙∙∙∙∙∙∙∙∙∙∙∙∙∙∙∙∙∙∙∙∙∙∙∙∙∙∙∙∙∙∙∙∙∙∙∙∙∙∙∙∙∙∙∙∙∙∙∙∙∙∙∙∙∙∙∙∙∙∙∙∙∙∙∙∙∙∙∙∙∙∙∙∙∙∙∙∙∙∙∙∙∙∙∙∙∙∙∙∙∙∙∙∙∙∙∙ 6
6. Basidiospores 14–17 × 6–7 μm ∙∙∙∙∙∙∙∙∙∙∙∙∙∙∙∙∙∙∙∙∙∙∙∙∙∙∙∙∙∙∙∙∙∙∙∙∙ *B*. *dissiliens*
  Basidiospores larger (16–26 × 6–11 μm according to Pegler & young 1981) ∙∙∙∙∙∙∙∙∙∙∙∙∙∙∙∙∙∙∙∙∙∙∙∙∙∙∙∙∙∙∙∙∙∙∙∙∙∙∙∙∙∙∙∙∙∙∙∙∙∙∙∙∙∙∙∙∙∙∙∙∙∙∙∙∙∙∙∙∙∙∙∙∙∙∙∙∙∙∙∙∙∙∙∙∙∙∙∙∙∙∙∙∙∙∙∙∙∙∙∙∙ *B*. *ananaeceps*
